# Associations between multi-method latent factors of puberty and brain structure in adolescent girls

**DOI:** 10.1016/j.dcn.2023.101228

**Published:** 2023-03-09

**Authors:** Michelle L. Byrne, Nandita Vijayakumar, Samantha J. Chavez, John C. Flournoy, Theresa W. Cheng, Kathryn L. Mills, Marjolein E.A. Barendse, Arian Mobasser, Jessica E. Flannery, Benjamin W. Nelson, Wen Wang, Elizabeth A. Shirtcliff, Nicholas B. Allen, Jennifer H. Pfeifer

**Affiliations:** a Turner Institute for Brain and Mental Health, School of Psychological Sciences, Monash University, Clayton VIC, Australia; b Department of Psychology, the University of Oregon, Eugene, OR, USA; c Deakin University, Centre for Social and Early Emotional Development, School of Psychology, Faculty of Health, Geelong, VIC, Australia; d Department of Psychology, Harvard University, Cambridge, MA, USA; e Psychiatric & Neurodevelopmental Genetics Unit, Massachusetts General Hospital, Boston, MA, USA; f PROMENTA Research Center, Department of Psychology, University of Oslo, Norway; g Department of Psychiatry and Behavioral Sciences, The University of California Davis, CA, USA; h University of North Carolina at Chapel Hill, Chapel Hill, NC, USA; i Centre for Adolescent Health, Murdoch Children's Research Institute, Parkville, VIC, Australia

## Abstract

Pubertal processes are associated with structural brain development, but studies have produced inconsistent findings that may relate to different measurements of puberty. Measuring both hormones and physical characteristics is important for capturing variation in neurobiological development. The current study explored associations between cortical thickness and latent factors from multi-method pubertal data in 174 early adolescent girls aged 10–13 years in the Transitions in Adolescent Girls (TAG) Study. Our multi-method approach used self-reported physical characteristics and hormone levels (dehydroepiandrosterone (DHEA), testosterone (T), and estradiol (E2) from saliva) to estimate an overall pubertal factor and for each process of adrenarche and gonadarche. There were negative associations between the overall puberty factor representing later stage and thickness in the posterior cortex, including the occipital cortices and extending laterally to the parietal lobe. However, the multi-method latent factor had weaker cortical associations when examining the adnearcheal process alone, suggesting physical characteristics and hormones capture different aspects of neurobiological development during adrenarche. Controlling for age weakened some of these associations. These findings show that associations between pubertal stage and cortical thickness differ depending on the measurement method and the pubertal process, and both should be considered in future confirmatory studies on the developing brain.

## 1 Introduction

Puberty is a sensitive period for neurobiological development, when major hormonal and physical changes play an important role in determining adolescents’ emotions and social behaviours via their actions on the developing brain (Dai and Scherf, 2019, Pfeifer and Allen, 2021, Vijayakumar et al., 2018a). While early theories proposed only “activational” effects of pubertal hormones, emerging animal and human research suggests that these hormones also exert “re-organizational” effects on the brain (Schulz et al., 2009), highlighting the importance of examining the role of puberty in structural neurodevelopment. Correctly measuring and operationalizing pubertal processes is important for capturing variation in both normative brain development and trajectories associated with adverse outcomes (Mendle et al., 2019). Crucially, puberty is a better predictor of many socioemotional outcomes than chronological age (Ullsperger and Nikolas, 2017). It is essential for developmental scientists who wish to relate pubertal and neural development to first understand how common methods of assessing puberty capture and often conflate different pubertal processes. The current study undertakes such an investigation in early adolescent girls, exploring how different pubertal processes are related to brain structure.

### 1.1 Pubertal processes and neurodevelopment

There are at least two processes within puberty that are activated and controlled by separate mechanisms (Counts et al., 1987). Adrenarche is the earlier phase of puberty that is triggered by the maturation of the zona reticularis of the adrenal gland around five to seven years of age (Parker et al., 1978, Remer et al., 2005). This leads to the release of adrenal hormones, such as testosterone (T) and dehydroepiandrosterone (DHEA) in girls, that are eventually responsible for secondary sex characteristics, such as pubic hair growth, body odour and acne (Parker et al., 1978, Rainey et al., 2002, Remer et al., 2005). Gonadarche is the later phase of puberty that is associated with maturation of the hypothalamic-pituitary-gonadal axis. The hypothalamus releases gonadotropin-releasing hormone that triggers the pituitary to produce follicle stimulating and luteinizing hormones, which in turn stimulate the gonads to produce sex steroid hormones (E2 in girls) that are responsible for reproductive maturity and other secondary sex characteristics (such as breast/genital development). Pubertal processes in developmental cognitive neuroscience are commonly measured based on self- or parent-report of observable physical characteristics, such as breast/genital development, pubic hair growth, skin changes, and body odour. Although a small subset of studies tease apart physical changes related to adrenarche and gonadarche (Hu et al., 2013, Vijayakumar et al., 2021a), most choose to collate across these two processes to examine overall pubertal maturity. Comparatively, examination of pubertal hormone concentrations may provide more specific insight into the endocrine processes that contribute to brain development.

Current neuroimaging research has predominantly examined associations between the physical changes during puberty and brain structure. These studies have focused on overall pubertal maturity, identifying a general pattern of reductions in cortical grey matter thickness with advancing pubertal stage (Koolschijn et al., 2014, Okada et al., 2020, Peper et al., 2009, Pfefferbaum et al., 2016, Vijayakumar et al., 2021a). However, convergence across studies in the location of these effects is limited. While many studies have reported significant associations in the frontal cortex, the specific region often varies (e.g., inferior frontal, (Koolschijn et al., 2014); cingulate and OFC, (Okada et al., 2020). Effects have also been noted outside the frontal cortex, including occipital (Herting et al., 2015) and parietal (Ando et al., 2021, Vijayakumar et al., 2021a) regions. We speculate that differences in correction for multiple comparisons, and relatedly, the choice of region-of-interest vs whole-brain analyses may be partly accountable. For example, some have reported significant associations in the frontal cortex when conducting region-of-interest but not whole brain analyses (Koolschijn et al., 2014), and others have noted significant associations only when lowering the stringency of correction for multiple comparisons in whole brain analyses (Peper et al., 2009). Others have reported significant associations in the frontal cortex using lobar measures without correction for multiple comparisons (Pfefferbaum et al., 2016). Thus, our understanding of pubertal effects on the cortex may be hindered by these varied analytic choices and a more exploratory study looking at brain-wide effect sizes using comprehensive measures of puberty is essential at this stage, in order to more accurately inform future confirmatory work.

While studies on hormones have more directly interrogated the role of adrenarche and gonadarche in female brain development, results are considerably varied (for an overview, see Fig. 1 in Vijayakumar et al., 2018a). E2 and T concentrations have typically been found to be negatively associated with cortical volume and thickness (Bramen et al., 2012, Brouwer et al., 2015, Neufang et al., 2009), although some have identified differential patterns across the cortex (Peper et al., 2009). Interestingly, research has most consistently reported association with T and structure of the posterior (parietal and occipital) cortices (Bramen et al., 2012, Koolschijn et al., 2014, Neufang et al., 2009, Nguyen et al., 2013). Only one study using a whole brain approach identified such effects in the frontal cortex (Bramen et al., 2012). When considering E2, some have reported widespread effects (Peperr et al., 2009) while others have failed to identify any significant effects (Koolschijn et al., 2014) when examining across the cortex. Others have reported effects within the middle temporal gyrus (Herting et al., 2015) or frontal and parietal cortices (Brouwer et al., 2015). Finally, little research has examined associations between DHEA and cortical thickness, with one study reporting associations in the temporoparietal and paralimbic cortices (Nguyen et al., 2013), but another failing to identify any significant effects (R. Ellis et al., 2019). Thus, extant literature has produced varied findings, which is likely attributable to varied analytic strategies (e.g., focus on specific regions vs the whole brain, and differences in methods of correcting for multiple comparisons). Much remains to be understood about the role of adrenarche and gonadarche in structural brain development. A comprehensive characterization of effect sizes across the cortex is warranted.

**Fig. 1:**
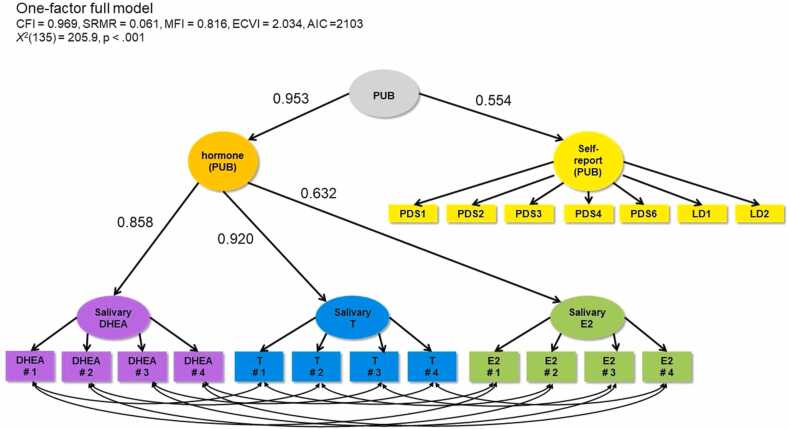
One-factor full model combining salivary DHEA, T, E2, and self-report items (PDS and LD). PDS1 = growth in height; PDS2 = growth of body hair (underarm and pubic); PDS3 = skin changes, especially pimples; PDS4 = breasts begun to grow; PDS6 = begun to menstruate (Y/N); LD1 = breast development (line drawing); LD2 = pubic hair (line drawing).

### 1.2 Multi-method measurement of pubertal processes

As discussed above, researchers can measure pubertal status with different methods such as pubertal hormones (i.e., DHEA, T, and E2) as well as with physical characteristics (e.g., breast/genital development, pubic hair growth and skin changes), either observed or self-reported from questionnaires like the Pubertal Development Scale (PDS; Petersen et al., 1988). Hormones have the advantage of being objective, “biological” measures that advance pubertal maturation. Including hormones in studies on brain development add an important sensitive continuous measure to a multi-method assessment of puberty. For example, we often only observe physical changes related to adrenarche, such as skeletal maturation and body odour, many years after hormone levels begin to increase (Wan, 2012). Research studies that only collect data on physical characteristics of puberty may therefore not have sufficient early indicators about this process. On the other hand, hormone measurements can be confounded by momentary and cyclical fluctuations. Further, measuring puberty with observable physical characteristics may additionally capture psychosocial mechanisms that influence neurobiological and behavioural development (Barendse et al., 2022). Thus, hormones and self-reported physical characteristics both provide unique information regarding pubertal processes, although we do not fully understand how each of these methods captures the underlying processes of adrenarche and gonadarche (i.e., latent factors of puberty) compared to variance in the measurement type. Understanding the latent structure of *multi-method* pubertal data may be useful for future research studies that attempt to understand both social and biological contributions of puberty to other outcomes such as psychopathology.

Despite a growing number of projects collecting both physical and hormonal indices of puberty, limited research has investigated how these measures can be incorporated into a multi-method assessment of puberty. One landmark study (Shirtcliff et al., 2009) found hormones and physical characteristics were generally correlated with one another. Another recent study modelled latent factors of pubertal hormones and physical characteristics from the Adolescent Brain Cognitive Development Study (Herting et al., 2021). However, while individual puberty measurements such as PDS scores have shown to be differentially associated with white matter microstructure in various brain regions (Herting et al., 2017), it is not yet clear if latent factors are associated with neurodevelopment, and if the items on the PDS and other measures of puberty fit best into a two-factor model of adrenarcheal and gonadarcheal processes, or if a model with one overarching factor of puberty is a better fit. In order to understand how pubertal processes may affect brain development, we must examine the contribution of multiple indicators of the dual processes of puberty and different measurements of puberty.

### 1.3 The current study

This investigation used multi-method cross-sectional pubertal data from a cohort of early adolescent girls aged 10–13 years to 1) develop a measurement framework of puberty for girls that includes multiple, commonly-used measures of puberty often used in developmental research while also accounting for how the processes of adrenarche and gonadarche fit into an overall model of puberty, and 2) explore covariances between these latent factors of puberty and cortical thickness in structural equation models (SEM). We focused on one biological sex because previous studies noted that correspondence between hormones and physical characteristics vary by sex (Shirtcliff et al., 2009).

First, we aimed to confirm a theory-driven approach by categorising self-reported physical development (measured by the PDS and Morris & Udry Line Drawings (LD); Morris and Udry, 1980), and biological sex hormones (measured in saliva and hair) into two expected latent factors: adrenarcheal and gonadarcheal development. We also compared the two-factor model to a one-factor model of general puberty to determine if common measurements of puberty can reliably differentiate between adrenarcheal and gonadarcheal processes. We considered adrenarcheal and gonadarcheal factors to capture the ongoing pubertal processes associated with adrenarche and gonadarche, i.e., originating in the adrenal gland and gonads, rather than the onset or timing of these developmental phases. Second, we explored the association between these latent factors of puberty and regional thickness across the cortical mantle. We focus on effect size estimation of these associations given inconsistencies in prior literature that may be driven by certain analytic choices (e.g., region-of-interest analyses and varied correction for multiple comparisons). The aim of the current study is exploratory in nature and will not be presented with significance testing (p-value) results. A careful and comprehensive exploratory approach, with a specific goal of hypothesis generation, is a critical step in developmental neuroscience to ensure robust and replicable confirmatory research in the future (Flournoy et al., 2020). Thus, the current cross-sectional investigation is aimed at presenting a comprehensive characterization of effect sizes that can serve as the foundation for subsequent longitudinal research and generate hypotheses to be tested in future waves of our project and studies in the broader field.

## 2 Method

### 2.1 Participants

We enrolled 174 participants from the community for the ongoing longitudinal study Transitions in Adolescent Girls (TAG; for protocol details, refer to (Barendse et al., 2020). Initially, 189 participants were recruited, but we excluded seven participants based on inclusion or exclusion criteria, and another eight withdrew before completing the initial assessment. The current analysis includes data from the first wave of the study, collected from 2016 to 2018, when the girls were 10.0–13.0 years of age (μ_age_ = 11.55 years, S.D. = 0.81). Models of latent pubertal factors were built from data from all 174 girls, and associations with brain structure were analysed in a subsample of 112 participants with high-quality structural MRI data, (see Section 2.3.1 for further detail).

### 2.2 Protocol

We obtained written informed consent from the parent/guardian and written assent from the adolescent. At this first visit, we trained the participants to collect their saliva samples by providing them with a saliva kit and instructions to provide one saliva sample per week, which were then completed at home. We also screened the adolescent for MRI eligibility as per procedures determined by the University of Oregon’s Lewis Center for Neuroimaging (LCNI).

At the second lab visit, the vast majority of which were scheduled approximately one month after their first visit, participants returned the saliva kit, completed questionnaires on pubertal development, and a researcher collected a sample of their hair. Except for the saliva samples, all measures in these analyses (MRI, hair, and questionnaires) were collected during the second session. Participants provided weekly saliva samples at home that were assayed for pubertal hormones. We compensated participants for their time, and the Institutional Review Board at the University of Oregon approved all materials and procedures.

### 2.3 Measures

#### 2.3.1 MRI assessment

We acquired data on a 3 T Siemens Skyra MRI scanner at the LCNI at the University of Oregon and collected a high-resolution T1-weighted structural image with the MP-RAGE sequence (TE=3.41 ms, TR=2500 ms, flip angle=7°, 1.0 mm slice thickness, matrix size=256 ×256, FOV=256 mm, 176 slices, bandwidth=190 Hz/pixel). We subsequently processed these images on a high performance computing cluster at the University of Oregon and performed cortical reconstruction using the FreeSurfer (v 6.0) image analysis suite (http://surfer.nmr.mgh.harvard.edu/), which provides tools for reconstructing topologically and geometrically accurate surface models of the inner and outer cortical boundaries, thus deriving anatomical measures such as cortical thickness.

Of the total sample of 174, 10 participants elected not to participate in the MRI. For the remaining 164 participants, trained researchers visually inspected and rated the quality of the raw T1 images on a three-point scale (1 =good, 2 =okay, 3 =bad), focusing on the presence of Gibbs rings and blurring (or sharpness) of images. Two separate raters examined each participants’ data, and a senior researcher resolved inconsistencies in ratings. We excluded poorest quality images (i.e., rating of 3; N = 25) from analyses. In addition, two senior researchers re-examined images with a rating of 2 (N = 112) to determine inclusion. Of these, we deemed another 27 to have major motion-related issues and excluded them from analyses, leaving a final sample size of 112 participants that had either no motion or minor motion-related issues that were limited to one area. In addition, a single researcher visually examined the FreeSurfer processed images to ensure quality of the cortical reconstruction (i.e., accuracy of grey-white and grey-pial boundaries). We excluded no further participants based on failures of cortical re-construction and did no manual correction of processed images. The Desikan Killiney parcellation was extracted to obtain thickness estimates for 68 cortical structures (34 per hemisphere).

#### 2.3.2 Hormone measures

##### 2.3.2.1 Saliva collection

To obtain a stable estimate of DHEA, T, and E2, given that they cycle over days and weeks even prior to menarche (Zhang et al., 2008), participants provided weekly saliva samples at home, in between the two lab sessions; the majority of participants completed four saliva samples, but some provided up to seven samples if lab sessions were rescheduled. For this analysis, we used only the last four saliva samples given before the second session (days between first and last sample: μ = 21.39, S.D. = 6.13, max = 57). We instructed participants to collect the samples (2 ml each) via passive drool on weekend mornings upon awakening and prior to the consumption of food or tooth brushing. They stored each vial in a cooler bag with Techni-ice in the family’s freezer until they transported the cooler bag to the University of Oregon for the second lab visit, where we moved the samples to a − 80 °C freezer in the lab until they were shipped (overnight with dry ice) to the Stress Physiology Investigative Team (SPIT) lab at Iowa State University. Along with each saliva sample, participants completed a form that recorded the day and time of collection. They also noted if they were sick in the prior 24 h, or had taken any medications during the previous day.

##### 2.3.2.2 Hair collection

We collected approximately 100 mg of hair near the posterior vertex of the scalp, which was shipped at room temperature to the SPIT lab at Iowa State University and then assayed for DHEA, T, and E2 using the methodology described by (Wang et al., 2019). We assayed the 5 cm closest to the scalp, reflective of cumulative hormone levels from the prior 5 months (as hair grows approximately 1 cm per month). Participants also completed a brief survey about their hair, including whether they i) had curly hair, ii) had permed or colored their hair (along with relevant time frames), and iii) felt their hair was relatively sweaty; however, these variables were not significantly associated with levels of hormones in our sample, so we did not include these as covariates in the current analysis.

#### 2.3.3 Self-reports of pubertal maturation

Participants self-reported their Tanner Stage using Line Drawings (LD; Morris and Udry, 1980). They viewed line drawings corresponding to the five Tanner stages of breast and pubic hair development, and were asked to choose images that best reflected their own development. Participants also completed the Pubertal Development Scale (PDS; Petersen et al., 1988), which assesses height growth, body hair and skin changes, as well as breast development and menarche in females. It consists of five questions that are scored on a 4-point scale ranging from development “not yet started” to “seems complete,” (PDS items 1–4) with the only exception being the yes/no item on menarche (PDS item 6). We did not incorporate the additional PDS item related to social comparison (PDS item 5). All self-report items were specified as ordinal variables in the models unless using a maximum likelihood (ML) estimator (see below).

### 2.4 Data Analysis

#### 2.4.1 Processing and cleaning hormone data

The SPIT lab assayed all hormones in hair and saliva in duplicate using ELISA kits (www.salimetrics.com). For details hair and saliva assaying protocol and intra- and inter-assay coefficients of variation, see Supplemental Material. We log-transformed hair and saliva hormone concentrations prior to analysis due to positive skew and kurtosis. Finally, we winsorized outlier values that were greater than 3 standard deviations from the mean (Hair N: DHEA=1; T = 3, E2 = 1; Saliva N: DHEA=0, T = 7, E2 = 7) to the value at + 3 SD plus 0.01 pg/ml increments to maintain the ordinal value of the outliers.

#### 2.4.2 Statistical modelling

**Fig. 2:**
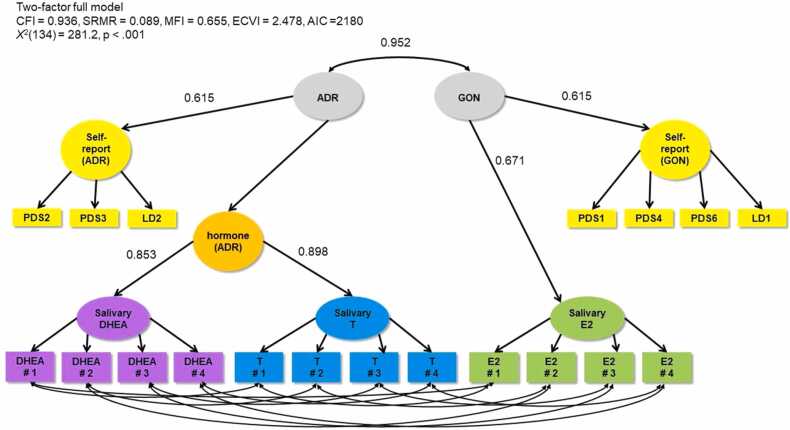
Two-factor full model combining salivary DHEA, T, E2, and self-report items (PDS and LD). PDS1 = growth in height; PDS2 = growth of body hair (underarm and pubic); PDS3 = skin changes, especially pimples; PDS4 = breasts begun to grow; PDS6 = begun to menstruate (Y/N); LD1 = breast development (line drawing); LD2 = pubic hair (line drawing).

We performed a series of confirmatory factor analyses (CFA) using the “lavaan” package (Rosseel, 2012) in R version 3.5.0 (Core Team, 2018) in three categories: 1) self-report models, 2) hormone models, and 3) full (i.e. combined self-report and hormone) models. Within each category, we noted if a two-factor (adrenal and gonadal; referred to as ADR and GON, respectively) or one-factor (pubertal; PUB) model provided better fit. We then compared the fit of models that did or did not include covariances between items within the same measure (i.e., PDS items,^2^ LD items, saliva days, and hair). For model comparisons, we report the McDonald Fit Index (MFI; (McDonald, 1989), the comparative fit index (CFI), expected cross validation index (ECVI; Browne and Cudeck, 1989), standardised root mean squared residual (SRMR), and robust chi-squared tests (X^2^). We considered smaller X^2^, SRMR, and ECVI values (closer to 0) and greater MFI and CFI values (closer to 1) to represent better fit. Refer to Fig. 1, Fig. 2 for visual representations of the one- and two-factor full models. Diagrams indicate standardised loadings. Code for the statistical modelling for this project is available on GitHub (Byrne et al., 2022).

Next, we conducted a series of SEM models that included covariances between regional cortical thickness and latent puberty factors (separately for each region). We chose not to examine nonlinear associations with brain structure given the cross-sectional nature of the data.

#### 2.4.3 Missingness

For initial SEM models that included ordinal data (self-report models), we used a pairwise maximum likelihood method (PML) to estimate parameters due to missing observations, to account for the ordinal nature of the PDS and LD items (and binary PDS item 6, menarche). For models with only continuous data (hormone models without any self-report indicators), we used maximum likelihood estimation (MLR) with robust (Huber-White) standard errors. The larger, full models were estimated with full information maximum likelihood estimators for missing data, as results were not substantially different between initial (i.e., separate self-report and hormone only) ML and PML models. For counts and percentages of missing data for the SEM models, please see Supplemental Table 1.

## 3 Results

### 3.1 Descriptive statistics of pubertal measures

To describe the sample’s pubertal status, we present means and standard deviations of several pubertal measures in Table 1. The self-report measures include the composite scoring using the PDS and LD, which uses relevant theory-driven items on those measures (based on Shirtcliff et al., 2009) to create an adrenarcheal (LD pubic hair and PDS adrenarcheal items; “Adrenarche composite”) and a gonadarcheal (LD breast development and PDS gonadarcheal items’ “Gonadarche composite”) score, as well as an average overall pubertal stage composite score (“Puberty composite”). Using the Shirtcliff et al. (2009) method, we also derived an overall “PDS stage” approximating Tanner stages from PDS data only. The hormone measures are mean values (non-transformed) of salivary (across four samples) and hair hormones (DHEA, T, and E2).

**Table 1:** Means and standard deviations of pubertal measures. DHEA = Dehydroepiandrosterone; E2 = Estradiol; PDS = Pubertal Development Scale; T = Testosterone.

Measure	Mean ± SD
Adrenarche composite (1–5)	2.88 ± 1.07
Gonadarche composite (1–5)	2.95 ± 0.91
Puberty composite (1–5)	2.91 ± 0.90
PDS stage (1–5)	2.95 ± 0.98
Salivary mean DHEA (pg/mg)	104 ± 127
Salivary mean T (pg/mg)	40.4 ± 24.1
Salivary mean E2 (pg/µg)	0.91 ± 0.55
Hair mean DHEA (pg/mg)	15.24 ± 14.84
Hair mean T (pg/mg)	1.81 ± 2.88
Hair mean E2 (pg/µg)	37.80 ± 15.82

### 3.2 Latent factor analysis

#### 3.2.1 Separate self-report and hormone models

We first explored one- and two-factor models, i.e., a single puberty factor versus separate adrenarche and gonadarche factors, with and without residual covariances using just the self-reported physical maturation data (PDS and LD; Supplemental Fig. 1). As models with and without residual covariances were equivalent (i.e., using a correlated uniqueness model to account for additional variance due to questionnaire did not improve model fit), residual covariances are not included in subsequent models of self-report data. Next, we explored one- and two-factor models with and without residual covariances with just the salivary and hair hormone data. However, there were low standardised loadings for observed hair DHEA, E2, T variables (consistently ≤ 0.4) on the latent puberty factors, even after constraining hair DHEA loadings to 0 as it was initially negatively estimated. Therefore, we tested additional *post hoc* models, with and without covariances, using only saliva variables, as shown in Supplemental Fig. 2. The one- and two-factor models for hormone only models are equivalent because there is only one indicator for the gonadal factor (E2) without hair. Adding residual covariances for the saliva hormone model between saliva samples collected on the same day improved fit (X^2^[12] = 143.0, p < 0.001) and fit indices were better. This suggests that covariances for biospecimens collected on the same day should be included.

Furthermore, we explored the covariance of the multiple saliva samples per hormone, and given that this covariation could change depending on pubertal stage, repeated these individual salivary hormone models separately for samples where Tanner stage from the PDS was > = 3, and where Tanner stage was < 3. In the full sample, DHEA loadings were 0.891–0.940; testosterone loadings were 0.715–0.872; estradiol loadings were 0.712–0.901. These loadings did not change substantially for each subsample of high and low Tanner stage participants, although fit indices were better in the low Tanner stage for DHEA and T. Diagrams of these models with individual loadings per sample are in Supplemental Table 3.

#### 3.2.2 Multi-method models of puberty

Our full one- and two-factor models (Fig. 1, Fig. 2 respectively) included all self-report and hormone variables, except hair hormones given the results of hormone models above, and included covariances for biospecimens collected on the same day. We also included second level latent variables for self-report (self-report ADR and self-report GON or self-report PUB). Fit indices favoured the one-factor model but were similar. Using the more parsimonious one-factor model, the shared variance between the hormonal latent puberty factor and the self-reported latent puberty factor was (0.953 ×0.554)^2^ = 30% indicating that there is considerable unshared variance. In the two-factor solution, the shared variance between the adrenal and gonadal factors was 0.952^2^ = 90%.

### 3.3 *Post hoc* exploration of age in the puberty models

**Fig. 3:**
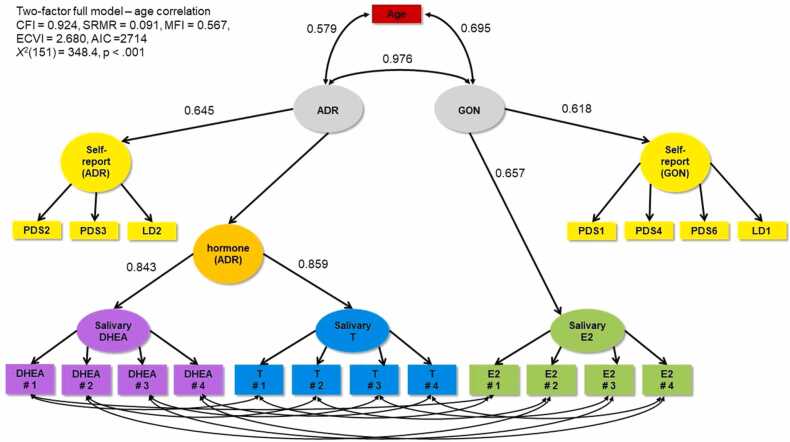
Two-factor full model correlating ADR, GON, and chronological age. PDS1 = growth in height; PDS2 = growth of body hair (underarm and pubic); PDS3 = skin changes, especially pimples; PDS4 = breasts begun to grow; PDS6 = begun to menstruate (Y/N); LD1 = breast development (line drawing); LD2 = pubic hair (line drawing).

**Fig. 4:**
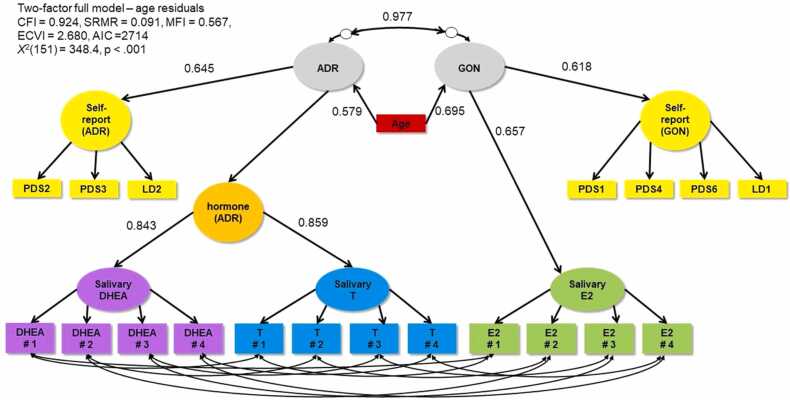
Two-factor full model correlating ADR and GON both controlling for age. PDS1 = growth in height; PDS2 = growth of body hair (underarm and pubic); PDS3 = skin changes, especially pimples; PDS4 = breasts begun to grow; PDS6 = begun to menstruate (Y/N); LD1 = breast development (line drawing); LD2 = pubic hair (line drawing).

Given there was some variation in the chronological age of the sample (range 10.0–13.0 years, μage = 11.55 years, S.D. = 0.81), and age is associated with pubertal development, we explored if age was a factor related to the ADR and GON latent factors that explained the positive correlations between those factors. First, we included age in the full model and allowed it to correlate with both the ADR and GON factors (Fig. 3), and found that it was significantly associated with ADR at r = 0.579 (p < 0.001, 34% shared variance) and GON at r = 0.695 (p < 0.001, 48% shared variance) but that ADR and GON still correlated at r = 0.976 (p < 0.001, 95% shared variance). Second, we fit a model that controlled for age (via ADR and GON factor regressions on age), to assess if correlations were due to variation in pubertal timing, which is stage compared to same-age peers (Fig. 4). ADR and GON were still correlated at r = 0.977 (p < 0.001, 95% shared variance). Loadings for ADR and GON onto their respective self-report and hormone factors were similar when including age in the model.

**Fig. 5:**
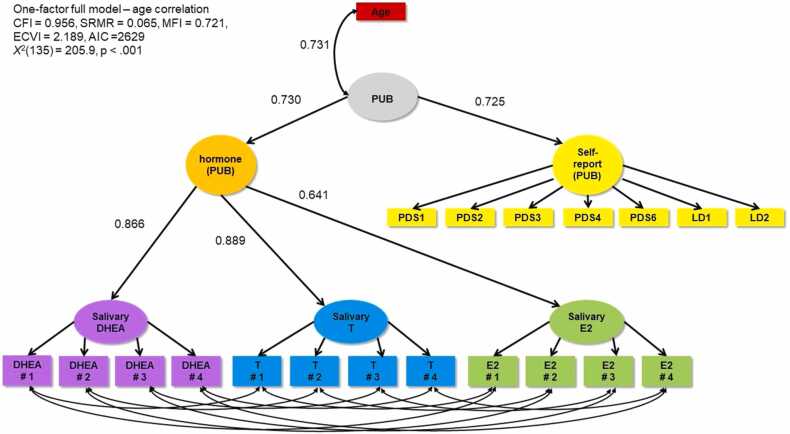
One-factor full model correlating PUB and chronological age. PDS1 = growth in height; PDS2 = growth of body hair (underarm and pubic); PDS3 = skin changes, especially pimples; PDS4 = breasts begun to grow; PDS6 = begun to menstruate (Y/N); LD1 = breast development (line drawing); LD2 = pubic hair (line drawing).

However, including age in the one-factor PUB model (Fig. 5), which showed significant association between age and PUB at r = 0.731, changed the loadings to be more similar across the hormone and self-report latent factors (non-age model: 0.953 correlation with hormones, 0.554 correlation with self-report; age model: 0.730 correlation with hormones, 0.725 correlation with self-report.

### 3.4 Inclusion of cortical thickness measures in models

**Fig. 6:**
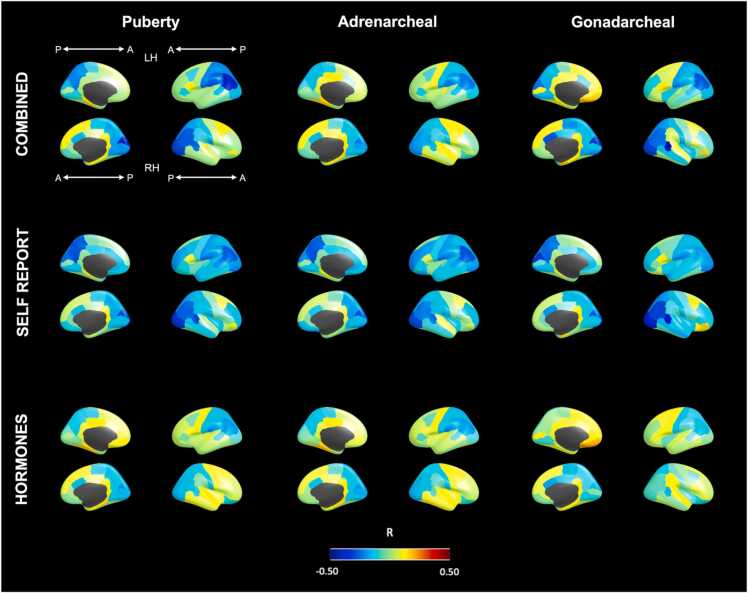
Estimated correlations from structural equation models between cortical thickness and multi-method (self-reported physical characteristics from the Pubertal Development Scale and Morris & Udry line drawing, and levels of hormone dehydroepiandrosterone, testosterone, and estradiol in saliva) latent factors of overall pubertal, adrenarcheal, and gonadarcheal stage in a sample of girls aged 10–13. Images were created with freesurfer_statsurf_display ( https://chrisadamsonmcri.github.io/freesurfer_statsurf_display). A: anterior; P: posterior; LH: left hemisphere, RH: right hemisphere, R: correlation coefficient estimated in latent models.

We conducted a series of SEM models that included covariances between regional cortical thickness and latent puberty factors. Using our multi-method latent models of puberty, the strongest associations were found to be negative, such that more advanced pubertal stage was related to thinner cortices (refer to Fig. 6 for full brain maps of these correlations). The overall puberty factor was most strongly correlated with thickness in the posterior cortices, with correlation coefficients between 0.3 and 0.4 present in the occipital lobe and extending laterally to the parietal lobe. Specific correlations for the right cuneus, right lateral occipital, and left inferior parietal are presented in Supplemental Table 2. A similar pattern of associations was identified for the gonadarcheal latent factor, in addition to medium sized effects within the right banks of the superior temporal sulcus (*R* = 0.43) and caudal anterior cingulate (*R* = 0.30). Comparatively, the adrenarcheal puberty factor was more weakly related to regional thickness, with the strongest associations being small effects (*R*s up to 0.27) in the posterior cortices.

We also repeated these analyses separately for self-report and hormone latent factors of puberty. As evident in Fig. 6, weak cortical associations were present for overall puberty, adrenarcheal and gonadarcheal latent factors from hormone models. Comparatively, stronger associations were present for all three latent factors from self-report models. Overall puberty and gonadarcheal factors exhibited a similar pattern of association in the self-report and the combined multi-method models, while the adrenarcheal factor exhibited stronger associations in the self-report relative to multi-method models.

Given that the puberty models presented above, especially the one-factor PUB model, changed due to the inclusion of age, we also conducted these models again for each region, allowing age to correlate with either PUB or with ADR and GON. For regions identified in the posterior cortices mentioned above (also included in Supplemental Table 2), correlations were somewhat weaker for the self-report latent factors for all identified regions and for all pubertal processes. For the latent hormonal factors, however, these correlations were much reduced and in some regions (e.g., parahippocampal and mid/posterior cingulate cortices), showed positive correlations between puberty and cortical thickness when holding age constant. Nevertheless, for the overall PUB multi-method (self-report and hormones combined), some correlations were stronger (e.g., left inferior parietal *R* = −0.419, right inferior parietal, *R* = −0.457, right cuneus, *R* = −0.420. These correlations are visually represented in Supplemental Fig. 4.

## 4 Discussion

This study of multi-method pubertal variables collected from early adolescent girls suggests, firstly, that self-reported pubertal characteristics and hormone levels from saliva can estimate an overarching pubertal process as well as separate processes of adrenarche and gonadarche, with a few caveats. Secondly, we identified negative associations between pubertal stage and cortical thickness, but the strength of these associations depend on measurement method (self-report or hormones) and pubertal process (adrenarche or gonadarche).

### 4.1 Multi-method measurement of pubertal processes

We first briefly discuss models conducted separately for self report and hormones, prior to our combined multi-method model.

#### 4.1.1 Self report data

We found that adding residual covariances for questionnaire type did not improve model fit for either the one- or two-factor model, suggesting that latent factors likely capture variance due to underlying pubertal process constructs rather than questionnaire-specific method variance. This finding also suggests that items that are theorised to belong to separable pubertal processes can be used to study the effect of self-reported physical characteristics related to the biological processes of adrenarche and gonadarche in future puberty studies with girls of this age range, but can also be used to measure a unitary pubertal process, depending on the research question. Interestingly, we also found that the item about height growth (PDS1) had the lowest standardised loading. Future studies could consider removing PDS1 if adrenarche and gonadarche are specifically of interest, because this item may reflect a different process that may not be synchronous to adrenarche or gonadarche (i.e., there may be individual differences in pubertal *synchrony*; for review, see Mendle et al., 2019). For example, it may contribute to a third somatotropic/growth axis process and the overall process of puberty, which may also be of interest to developmental science. The growth hormone/insulin-like growth factor 1 (GH-IGF-1) axis is triggered by an increase in pubertal hormones such as E2 during puberty (Christoforidis et al., 2005), and also contributes to the onset of puberty via the release of gonadotropin-releasing hormone (Dees et al., 2021). However, the growth axis represents a separate developmental process to adrenarche and gonadarche. For example, in one study of adolescent girls, while a significant correlation between DHEA and IGF-1 in younger (prepubertal) girls was found, these hormones did not correlate in older girls (Guercio et al., 2003). This is aligned with the current study’s findings in the self-report data, showing that the PDS1 item had the lowest loading on latent self-report factors of puberty, including gonadarche. Longitudinal studies would benefit from examining the growth axis more carefully over time within individuals by including the PDS1 item and other self-reported measures of the growth axis in addition to GH-IGF-1.

#### 4.1.2 Hormone data

Initial hormone models conducted with saliva and hair models did not fit well. This may be because we had more salivary observed measures (four) than hair (one). Also, the duration over which we measured saliva and hair in this sample differed. The four saliva samples were measured over approximately one month and combined into one latent factor. However, the single observed hair sample reflected levels of hormones that were aggregated over approximately five months of hair growth. In previous research with adults, more saliva samples were needed in order for hormone levels to significantly correspond with hair levels (Wang et al., 2019). Future research should collect saliva and hair over similar time periods in order to more accurately compare levels of pubertal hormones between the two biospecimens.

Removing hair hormones improved model fit indices. Further, including separate covariances for each day of saliva collection and for all observed hair variables significantly improved fit for the one- and two-factor models, suggesting that these account for some of the biospecimen variance independent of puberty.

#### 4.1.3 Full latent factor models

In models that included all variables (except hair hormones), a more parsimonious one-factor model had better fit indices than the two-factor model. We therefore favour the more parsimonious one-factor model of puberty statistically, but separate latent factors of adrenarcheal and gonadarcheal processes can also be used *if research questions about those mechanisms are justified*. In other words, researchers are justified in loading multi-method biological and self-reported puberty data from girls aged 10–13 years onto an overarching puberty factor, or separate adrenarche and gonadarche factors. The generalizability of this structure is yet unknown in other populations.

The shared variance between the hormonal latent puberty factor and the self-reported latent puberty factor was 30%. While this amount of concordance between hormones and self-report puberty data is higher than previous studies that measured correspondence between salivary hormones and self-reported puberty (e.g., Grotzinger et al., 2018, Shirtcliff et al., 2007, Shirtcliff et al., 2009), it also suggests that measuring puberty via hormones and via self-report questionnaires is not redundant (68% not shared variance), and models of puberty would benefit from collecting both. This was also apparent in the two-factor model, where for the ADR factor especially, loadings for the self-report and saliva factors were not completely similar. Puberty is not a singular event or process, and it is important to remember that including multiple measures and processes may be more representative of the “true score” of pubertal processes, regardless of the correspondence between biospecimens and self-report questionnaires.

### 4.2 Correlations with brain structure

We examined the relationship between pubertal stage and brain structure first for the one-factor model of puberty that combined self-reported physical characteristics and hormones. Findings revealed the strongest negative associations in the posterior cortex, including the occipital cortices and extending laterally to the parietal lobe. This is consistent with the overall *direction* of negative associations between puberty and cortical thickness that have typically been identified in prior literature (Koolschijn et al., 2014, Okada et al., 2020, Pfefferbaum et al., 2016, Vijayakumar et al., 2021b). However, the *location* of strongest effects within posterior cortices differs from prior findings that have most typically implicated frontal regions (Koolschijn et al., 2014, Okada et al., 2020, Pfefferbaum et al., 2016, Vijayakumar et al., 2021b). Interestingly, this is consistent with prior suggestions of earlier age-related maturation of sensory relative to higher-order cognitive systems (Grayson and Fair, 2017, Zielinski et al., 2010). Given that we identified a similar pattern of pubertal effects when accounting for age (discussed below), this raises the possibility that pubertal processes may also be implicated in earlier maturation of posterior sensory systems. Finally, although we chose to present a comprehensive characterization of effect sizes (rather than significance testing), the strength of associations in the posterior cortex (∼0.3) was comparable in size to prior research that has reported significant correlation coefficients between puberty and brain structure (Blanton et al., 2012, Nguyen et al., 2013, Peper et al., 2009).

Examining the two gonadarcheal and adrenarcheal factors separately revealed a similar pattern of cortical associations to the one factor model, with the strongest associations in the posterior cortex. Interestingly, the multi-method approach resulted in weaker cortical correlations for adrenarche relative to gonadarche. To understand whether these differences were driven by measurement approaches, we also examined cortical associations for self-report and hormone measures separately. Findings suggest that there may be overlap of the shared variance of certain cortical structures with both gonadarcheal physical characteristics and hormones, which leads to stronger effects in the multi-method (combined) approach. Comparatively, there may be less overlap of shared variance between cortical structures and both adrencheal physical changes and hormones, leading to weaker effects in the multi-method approach. As an example, we tease apart results for the left inferior parietal cortex. Self-report and hormone gonadarcheal factors were correlated with thickness at − 0.23 and − 0.15, respectively, while stronger correlations for the multi-method gonadarcheal factor (−0.30) at suggests greater common variance leads to “boosting” of identified gonadarcheal processes. Conversely, self-report and hormone adrenarcheal factors were correlated with thickness at − 0.30 and − 0.23, respectively, while weaker correlations for the multi-method factor (−0.27) suggests less common variance and potentially differential effects of adrenarcheal physical changes and hormones on the brain. In other words, we speculate that these differences may reflect varied mechanisms of brain development captured by different measures for gonadarche vs adrenarche. Further research into these processes is required to understand when a multi-method approach is most advantageous in puberty research. Self-report and hormonal indices of pubertal development can be complementary both by providing more robust measurement of pubertal development (broadly), but also by providing unique windows into psychosocial and biological mechanisms of pubertal change in humans when examined separately. Crucially, this method may be better powered to identify pubertal processes in certain ages, with implications for research into the social, cognitive and emotional outcomes of this period.

While latent factors from hormone models exhibited overall weak correlations with brain structure, the strongest negative associations remained in the posterior parietal and occipital cortices (e.g., the association between left inferior parietal cortex and both adrenarcheal and overall puberty stage factors was −0.23). As outlined in the introduction, prior research has also most consistently identified negative associations between testosterone and thickness of posterior cortices, including the precuneus, superior and inferior parietal, calcarine and lingual cortices (Bramen et al., 2012, Koolschijn et al., 2014, Neufang et al., 2009, Nguyen et al., 2013). However, we note that some other regions that have been previously implicated had weak effects in the current study (for an overview of these associations, see Fig. 1 in Vijayakumar et al., 2018b). This was particularly evident for associations between estradiol and thickness. While prior research has identified varied locations of significant associations between estradiol and thickness (Brouwer et al., 2015, Herting et al., 2015, Peper et al., 2009), we failed to identify any moderate or strong effects. We speculate that these differences are likely driven by the varied analytic strategies used to examine hormonal associations with brain structure (e.g., choice of regions of interest).

#### 4.2.1 Controlling for age

Holding age constant, the strength of negative associations was reduced between latent pubertal factors and thickness in posterior cortices for self-report (across puberty, adrenarcheal and gonadarcheal factors). Controlling for age also resulted in more positive associations for hormones (again across all factors), with strongest effects evident in the medial orbitofrontal as well as mid- to posterior-cingulate extending to parahippocampal cortices. These appear to be driven by the negative association between age and cingulate thickness. Indeed, others have reported positive associations between testosterone and the occipital cortices (in boys alone; Bramen et al., 2012), as well as estradiol and the occipital, anterior temporal and dorsolateral prefrontal cortices (Peper et al., 2009) when controlling for age. Thus controlling for age may be removing some of the variance that we are interested in examining, as it does not present “normative” or group-level associations between brain structure and hormones. It instead reflects associations with hormone levels that are high/low for a given age or stage (i.e., pubertal timing).

Interestingly, the negative association between the overall puberty multi-method factor and some regions strengthened. The multimethod approach may be sensitive to negative associations driven by self-report as well as positive associations driven by hormones. Furthermore, increased strength of associations using the multimethod approach suggests sensitivity to shared variance across self-report and hormone measures, leading to a “boosting” of effect sizes (similar to findings without the age covariate). This highlights the importance of presenting results with and without these covariates, as well as the relationship between these covariates and brain structure. We emphasise the need for replication efforts that characterise the correlations across the brain in order for us to gain a clearer understanding of the role of pubertal hormones in brain development. We would argue that there is a need for continued research that focuses on effect sizes across the cortex, to improve our understanding of these developmental processes. Overall, this *post hoc* finding when controlling for age presents some preliminary evidence that pubertal *stage* (not controlling for age) and pubertal *timing* (controlling for age) may not have the same effects on the developing brain, so future confirmatory work is justified in focusing on different regions for each, depending on the specific research question, and even different directions of effects in some cases.

### 4.3 Strengths and limitations

We present a comprehensive study into the structure of puberty in early adolescent girls, using multiple methods of measuring hormones and self-reported physical characteristics. In addition, it is the first study to use this multi-method approach to examining puberty to see how it is related to cortical structure, considering separable processes of adrenarche and gonadarche. We focused on reporting effect sizes across all cortical regions due to inconsistencies reported in previous research that may have been obscured by corrections for multiple comparisons or only focusing on certain regions. However, the conclusions from the current exploratory study should be interpreted as preliminary findings, and should be used to inform new, confirmatory (and directional) hypotheses. For example, research questions that wish to examine associations in early adolescence between overall pubertal stage and cortical thickness may hypothesise negative associations with the posterior cortices, i.e., the occipital and parietal lobe, and may define regions of interest as the right cuneus, right lateral occipital, and left inferior parietal. Researchers with questions that focus on adrenarcheal or gonadarcheal effects are justified to create separate multi-method latent scores, but should also consider including physical exams in adrenarcheal models. On the other hand, when pubertal timing (models controlling for age) may be the predictor of interest, for example with mental health outcomes, then researchers may not expect the effects to be as strong, or in some regions (namely the parahippocampal and mid/posterior cingulate cortices), they may predict positive associations with puberty, controlling for age.

There are a few limitations to note for future research. First, the current sample is similar to the local population in Oregon, U.S, but has limits to its broader generalizability because of limited racial and ethnic diversity. There are significant individual differences in pubertal processes, including differences related to race, ethnicity, sex, gender, and socioeconomic status (B. J. Ellis, 2004). Measurement of puberty in research currently suffers from a lack of inclusion of low-income youth; youth with diverse sexual, gender, racial, and ethnic identities; and boys (Deardorff et al., 2019, Mendle et al., 2019). Also, results from samples measured at other ages during puberty (earlier at the start of adrenarche, or later when there is more variability in gonadal processes) may differ; that is, we may find more differentiation of the two factors at different ages. We encourage future research to replicate our structural organisation of multi-method puberty data with samples from other populations, especially those from large-scale, representative studies. This may include publicly available data from studies like the Adolescent Brain Cognitive Development (ABCD) study, although it should be noted that smaller-scale studies, like the current study, often have more comprehensive measures of puberty. For example, ABCD data does not include line drawings of Tanner stages or more than one saliva sample per wave (Cheng et al., 2021). We need both types of studies to understand the associations between pubertal and neural development.

Second, our analyses were cross-sectional because our aim was to organise indicators of pubertal stage during a snapshot of development. It is not possible to comment on developmental trajectories with this cross-sectional sample, and longitudinal intra-individual analyses are needed to corroborate our findings. We also did not examine nonlinear associations between puberty and brain structure as longitudinal research is most suited to examine such developmental trends. For this reason, we did not incorporate subcortical structures (that most typically exhibit nonlinear trajectories; (Goddings et al., 2014, Vijayakumar et al., 2021, Wierenga et al., 2018) into our analyses. Furthermore, volume is a product of thickness and surface area, and combining these two properties can obscure characteristics that are unique to each structural property (Panizzon et al., 2009). We restricted our analyses to cortical thickness that has most consistently been shown to exhibit linear patterns of reduction within our age range and thus we would be adequately powered to capture in our sample.

Third, these analyses/models did not include covariates, such as body mass index and early life stress that tend to exhibit associations with pubertal and brain development. We additionally note that controlling for the full set of social, genetic, and environmental factors that influence puberty and the brain is neither possible with this data (because we lacked comprehensive measures), nor is it necessarily desirable. Instead, we conceive of puberty and brain development as processes comprising social, genetic, and environmental components, and remaining variance after controlling for these factors may not be ecologically meaningful. The next step for puberty research is to use this structure to model individual differences in predictors and outcomes of puberty both cross-sectionally and with trajectories over time within subjects, in larger, better powered samples, examining not only pubertal stage, but timing and tempo, as well.

Fourth, additional measures of puberty that were not included in this study may contribute further to overall latent factors, or change the fit of the current measures. For example, physical exams conducted by clinicians were not used in our study. The previously mentioned Shirtcliff et al. (2009) study showed that while self-report measures such as the PDS and picture-based interview of puberty (PBIP) correspond well with a physical exam score, the PDS may still miss important aspects of adrenarche that happen earlier in development (Dorn, 2006). Therefore, future studies attempting to create latent adrenarcheal scores that capture all aspects of this pubertal process may benefit from including clinician exams.

Finally, there were a few possible confounders specifically in our biological data that may have influenced our results. Saliva samples were collected by the participant at home, and although we instructed them to collect the sample first thing in the morning, some variation in adherence to time of day of collection could have influenced results. However, we included covariances for saliva samples collected on the same day to account for any confounders that may have been due to anything particular about that sample day, including variation in time of day. Additionally, as mentioned previously, we measured an aggregated level of hormone in hair over five months whereas saliva samples taken over a duration of one month were combined into a latent factor, and this difference in duration could have affected our results.

## 5 Conclusions

In this multi-method study of pubertal indicators in a sample of early adolescent girls, we found that self-reported puberty data from the PDS and line drawings and salivary hormones (DHEA, T, and E2) all load well onto theorised latent factors of pubertal processes. Overall, we did not find differences in fit between models that included only one overarching pubertal factor and those that differentiated between adrenarcheal and gonadarcheal factors. However, differentiating these processes in an analysis with brain structure showed that associations between pubertal stage and cortical thickness differ depending on the pubertal process and the method of measurement. A multi-method latent factor of a unitary (overall) pubertal process showed associations in the posterior cortex, including the occipital cortices and extending laterally to the parietal lobe, but when examining adrenarcheal and gonadarcheal latent processes on their own, the multi-method approach for adrenarche had weaker cortical correlations. More work is needed to assess if hormones and self-reported physical characteristics are reflecting the same mechanisms in brain development during adrenarche. This represents an important avenue for future research, with implications for assessing and understanding the role of specific pubertal processes on the developing brain and related consequences for socioemotional outcomes during early adolescence.

## Declaration of Competing Interest

The authors declare the following financial interests/personal relationships which may be considered as potential competing interests: Michelle Byrne reports financial support was provided by National Institute of Mental Health. Theresa Cheng reports financial support was provided by National Center for Advancing Translational Sciences.

## Data Availability

Explanation is in the manuscript text.

## Footnotes

2 We did not include covariances for PDS6, a binary item that asks if menarche has begun, as we considered this to be a different measure than the other PDS items, which ask if a process is not yet started, barely started, definitely underway, or seems complete.
