# Supplementary material for: Associations between multi-method latent factors of puberty and brain structure in adolescent girls
Source: Dev Cogn Neurosci. 2023 Mar 9;60:101228. doi: 10.1016/j.dcn.2023.101228 (PMC10031110; doi:10.1016/j.dcn.2023.101228)
Supplement: Supplementary file 1 — Supplementary material [file mmc1.docx]

**Supplemental Material**

**Hair and saliva assay details and non-detectable values**

For hair, samples were washed, ground, extracted, reconstituted and assayed using methodology described by Wang et al. (2019); estradiol required a double-extraction to purify the sample prior to assay. We re-assayed saliva samples when the optical density intra-assay coefficient of variation (CV) was greater than 7%, if enough sample was left over to do so. We assayed all hormones for each participant on the same day to minimise freeze-thaw cycles, and on the same plate, so as to minimise variation in hormone concentrations that may be attributable to plate differences (i.e., inter-assay CVs). All CVs reported are for the optical density wavelengths. The intra-assay CVs for saliva were 10.48% for DHEA, 1.80% for T, and 7.76% for E2, and the intra-assay CVs for hair were 1.97% for DHEA, 2.70% for T, and 2.87% for E2. We processed the samples in two batches. For saliva, the inter-assay CVs for Batch 1 were 20.62% for DHEA, 10.23% for T, and 11.53% for E2, and for Batch 2 were 21.43% for DHEA, 8.34% for T, and 15.55% for E2. For hair, the inter-assay CVs for Batch 1 were 2.42% for DHEA, 8.45% for T, and 7.81% for E2, and for Batch 2 were 17.08% for DHEA, 0.72% for T, and there was only one plate for hair E2.

The number of mean salivary hormone concentrations (of the duplicates) that were non-detectable were as follows: DHEA: samples=38, participants=20; T: samples=3, participants=3; E2: samples=23, participants=13.

We substituted salivary hormone concentrations that were non-detectable and too low (i.e., left-censored; DHEA samples = 32, T samples = 3, E2 samples = 23) using the following rules:

1) If other samples from the participant (i.e. from other sampling days) were also not detectable, the mean of all samples were replaced with the lower limit of sensitivity (DHEA: 5 pg/ml; T: 1 pg/ml; E2: 0.1 pg/ml);

2) If other samples from the participant were detectable and 50% or more of the remaining samples were below the lower interval of the inter-quartile range (IQR) for the distribution, the mean of the non-detectable sample was replaced with the lower limit of sensitivity for that assay;

3) If other samples from the participant were detectable and less than 50% of the remaining samples were below the IQR for the distribution, (i.e., most of the remaining samples were detectable and higher than the bottom quartile), the mean of the non-detectable sample was considered missing.

For mean salivary hormone concentrations that were too high (i.e., right-censored above the upper limit of the standards for that assay; DHEA only, samples = 6), we applied the following rules:

1) If 50% or more of the remaining samples were above the upper IQR, the right censored sample was replaced with the upper limit of the standards for that assay (DHEA: 1000 pg/ml; T: 600 pg/ml; E2: 32 pg/ml).

2) If less than 50% of the remaining samples were above the upper IQR, the mean of the right censored sample was considered missing.

We substituted hair hormone concentrations (mean of the duplicates) that were non-detectable and right-censored with the upper limit of the standards for that assay (N: DHEA=0, T=2, E2=0). No mean hair hormone concentrations were non-detectable and left-censored.

**Missing Data**

After following rules for replacement of hormone concentrations above, Supplemental Table 1 summarises the counts and percentages of missing data.

| **Variable** | **Missing (Total N = 174)** | **Percentage missing** |
| --- | --- | --- |
| Salivary DHEA sample 1 | 11 | 6.32 |
| Salivary DHEA sample 2 | 11 | 6.32 |
| Salivary DHEA sample 3 | 16 | 9.20 |
| Salivary DHEA sample 4 | 26 | 14.94 |
| Salivary T sample 1 | 8 | 4.60 |
| Salivary T sample 2 | 9 | 5.17 |
| Salivary T sample 3 | 15 | 8.62 |
| Salivary T sample 4 | 26 | 14.94 |
| Salivary E2 sample 1 | 9 | 5.172 |
| Salivary E2 sample 2 | 9 | 5.172 |
| Salivary E2 sample 3 | 14 | 8.046 |
| Salivary E2 sample 4 | 25 | 14.37 |
| Hair DHEA | 26 | 14.94 |
| Hair T | 26 | 14.94 |
| Hair E2 | 66 | 37.93 |
| PDS1 (growth in height) | 11 | 6.32 |
| PDS2 (growth of body hair) | 12 | 6.90 |
| PDS3 (skin changes) | 9 | 5.17 |
| PDS4 (breasts begun to grow) | 13 | 7.47 |
| PDS6 (begun to menstruate) | 9 | 5.172414 |
| LD1 (breast development) | 17 | 9.770115 |
| LD2 (pubic hair) | 20 | 11.49425 |

***Supplemental Table 1****: Rates of missingness for all variables*

**Self-report only and hormone only models**

For self-report only models (PDS & LD; supplemental Figure 1) there was a small but not statistically significant difference between the one- and two-factor models (puberty vs. adrenarche and gonadarche), with the two-factor model fitting marginally better (X^2^(1) = 2.9, *p* = 0.090). Loadings for all items were higher on the two-factor model. Constraining the model to only one latent factor (PUB) did not fit the data worse. In other words, a more complex, two-factor model of puberty did not fit the self-report data better than the more parsimonious one-factor model.

For hormone only models (Supplemental Figure 2), the one and two factor models were equivalent but including covariances improved model fit, as discussed in the main text Results.

**
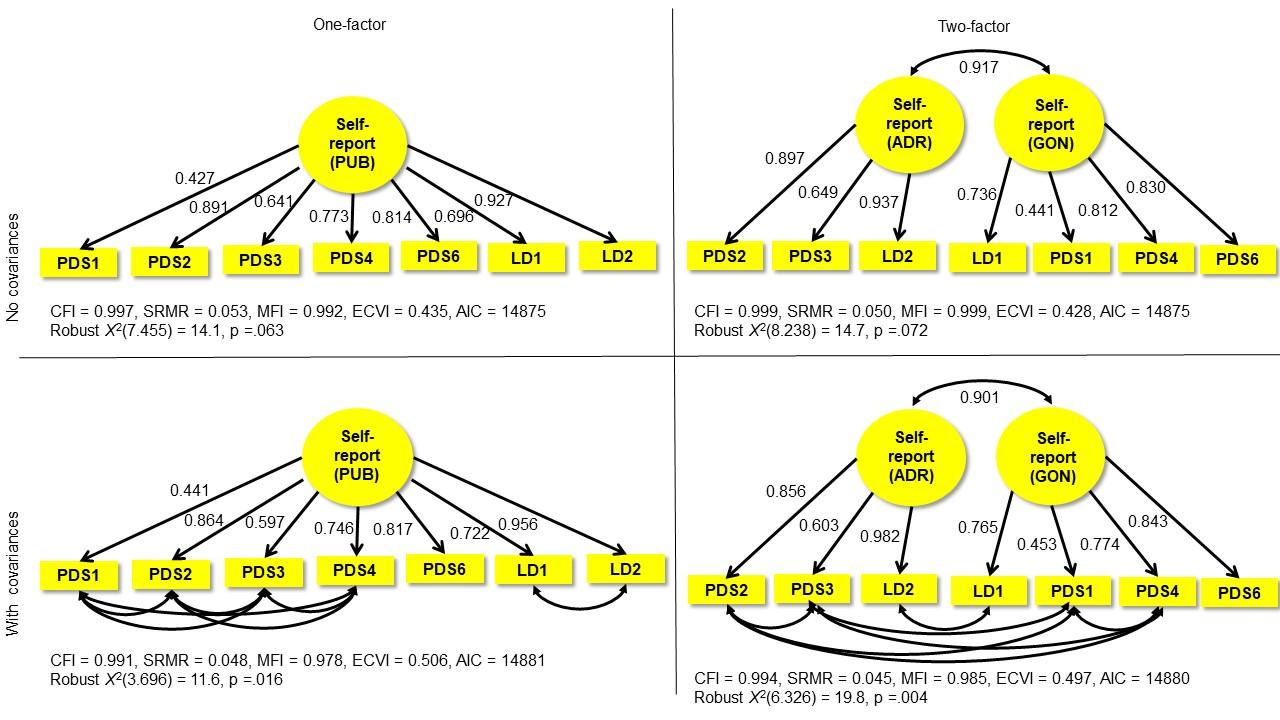
**

***Supplemental Figure 1****: One- and two-factor self-report models with and without covariances, including items from PDS and LD questionnaires. PDS1 = growth in height; PDS2 = growth of body hair (underarm and pubic); PDS3 = skin changes, especially pimples; PDS4 = breasts begun to grow; PDS6 = begun to menstruate (Y/N); LD1 = breast development (line drawing); LD2 = pubic hair (line drawing). Self-report items are ordinal except for PDS6, which is binary.*


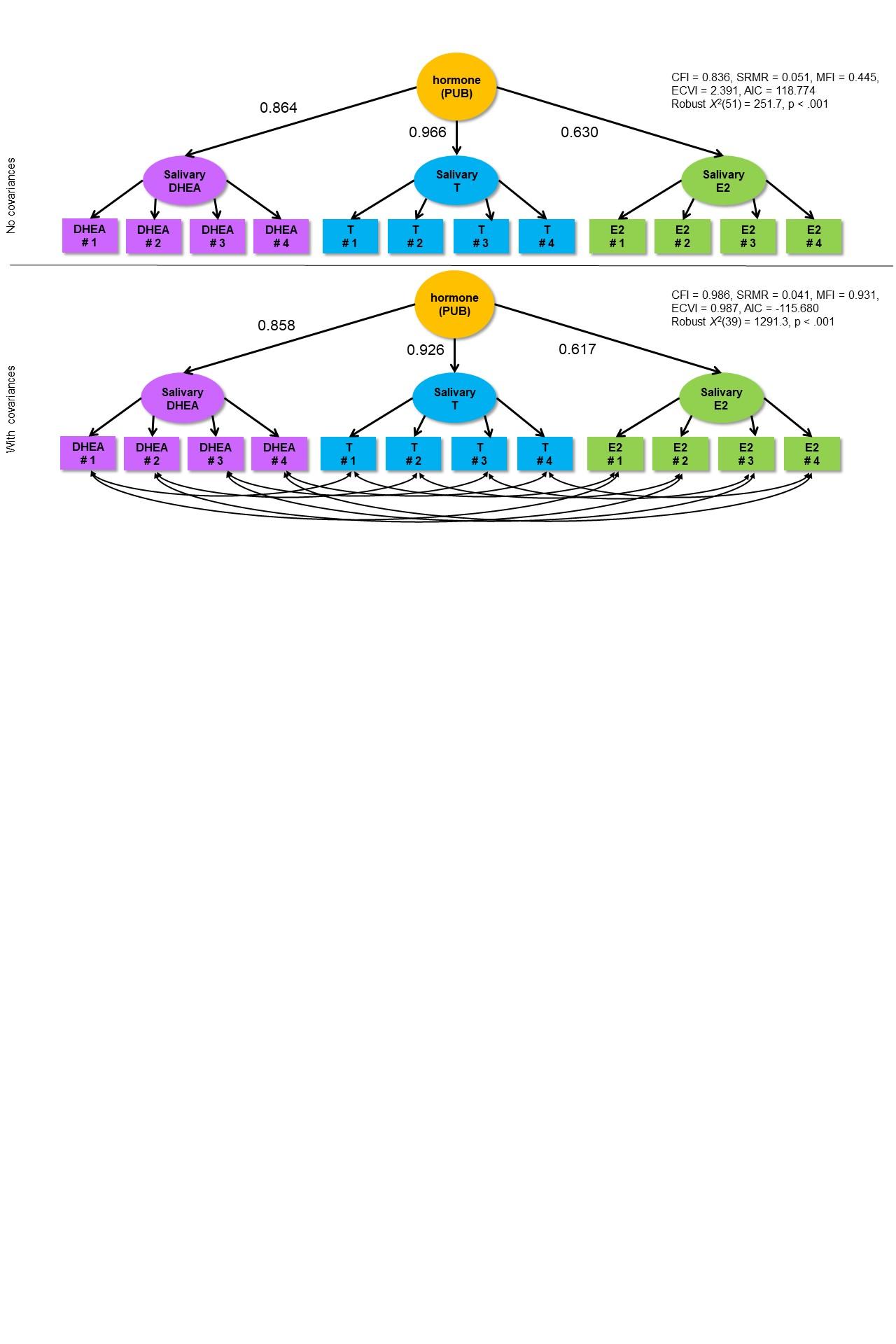


***Supplemental Figure 2****: Saliva only hormone models with and without covariances, including all salivary variables.*

**Covariation of multiple saliva samples**

We explored loadings of the four saliva samples for each hormone on their own, and also separately for subsamples with high (>= 3) or low (< 3) Tanner stage. Diagrams of these models with individual loadings are in Supplemental Figure 3.


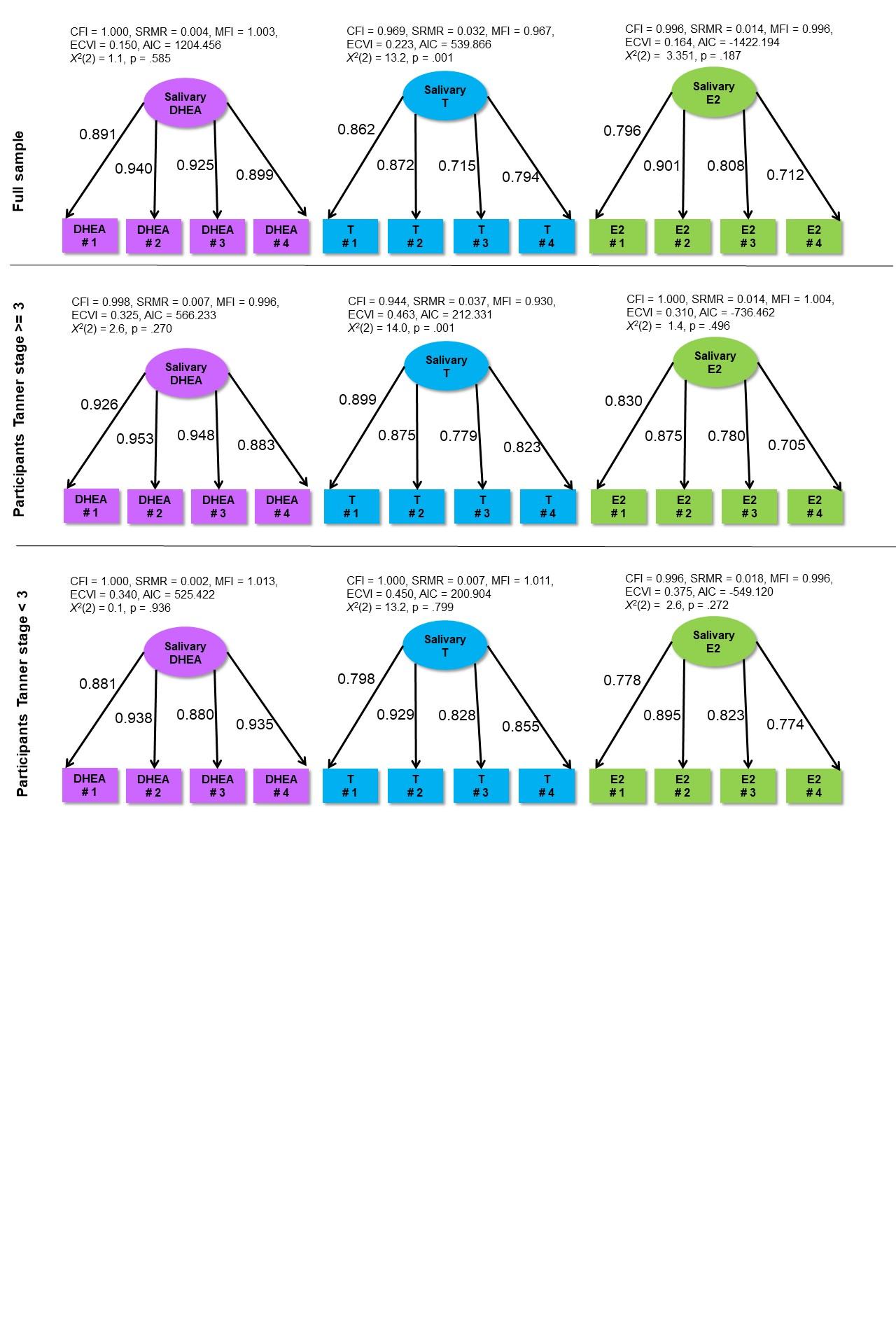


***Supplemental Figure 3****: Separate saliva hormone models with and without self-reported pubertal stage (latent) included. For indicators on Self-report (PUB), refer to Supplemental Figure 1*

**Latent vs. non-latent measures of puberty**

We identified three brain regions where the effect size of the association between thickness and the one-factor latent puberty variable was strongest: right cuneus, right lateral occipital, and left inferior parietal. We have also included correlations from models that included chronological age for comparison.

To demonstrate how creating a latent variable from puberty measures may be beneficial compared to only using traditional measures, we present in Supplemental Table 2 the correlations of self-report and hormone latent variables compared with a composite scoring using the PDS and LD, which uses relevant theory-driven items on those measures (based on Shirtcliff, et al., 2009) to create an adrenarcheal (LD pubic hair and PDS adrenarcheal items) and a gonadarcheal (LD breast development and PDS gonadarcheal items) score, as well as an average overall pubertal stage score. We also compared brain correlations with the latent factors to brain correlations with the mean value of the four samples of relevant salivary hormones (DHEA, T, and E2 for the overall pubertal score, DHEA and T for the adrenarcheal score, and E2 only for the gonadarcheal score). We also present correlations for the overall (multi-method) puberty factors. Correlations with all brain regions were stronger for latent variables compared to their self-report or hormone non-latent counterparts, with the exception of a few gonadarcheal correlations: self-report and left inferior parietal, and hormone and right cuneus and left inferior parietal. The gonadarcheal hormone latent correlations may be weaker because it only includes E2, and the latent model included additional complexity for the sample day covariances. Correlations from the full latent multi-method models were almost always stronger than from any single method for overall puberty and gonadarcheal processes (except for right lateral occipital), but weaker for adrenarcheal processes when full model is compared to self-report methods, as noted in the Discussion in the main text. Additionally, correlations between the self-report composite and age (PUB: 0.496; ADR: 0.494, GON: 0.406) were similar to correlations between the mean levels of hormones and age (PUB: 0.530; ADR: 0.497; GON: 0.450), also referred to in the Discussion.

|  | **Puberty** | | | **Adrenarcheal** | | | **Gonadarcheal** | | |
| --- | --- | --- | --- | --- | --- | --- | --- | --- | --- |
|  | Right cuneus | Right lateral occipital | Left inferior parietal | Right cuneus | Right lateral occipital | Left inferior parietal | Right cuneus | Right lateral occipital | Left inferior parietal |
| Latent (without age) self-report only | -0.311 | -0.326 | -0.298 | -0.305 | -0.293 | -0.309 | -0.292 | -0.357 | -0.231 |
| Self-report composite | -0.298 | -0.303 | -0.257 | -0.285 | -0.246 | -0.230 | -0.275 | -0.340 | -0.253 |
| Latent (with age) self-report | -0.231 | -0.225 | -0.240 | -0.227 | -0.209 | -0.240 | -0.227 | -0.242 | -0.213 |
| Latent (without age) hormone only | -0.225 | -0.141 | -0.230 | -0.219 | -0.143 | -0.230 | -0.161 | -0.081 | -0.147 |
| Mean salivary levels of hormones | -0.218 | -0.122 | -0.202 | -0.215 | -0.133 | -0.190 | -0.165 | -0.058 | -0.175 |
| Latent (with age) hormone | -0.156 | 0.015 | -0.137 | -0.154 | 0.013 | -0.137 | -0.080 | 0.062 | -0.057 |
| Latent (without age) full multi-method model | -0.367 | -0.302 | -0.369 | -0.257 | -0.187 | -0.269 | -0.343 | -0.305 | -0.302 |
| Latent (with age) full multi-method model | -0.420 | -0.255 | -0.419 | -0.219 | -0.063 | -0.206 | -0.264 | -0.078 | -0.243 |

***Supplemental Table 2****: Comparisons between cortical thickness in selected brain regions and latent vs. non-latent measures of puberty and multi-method latent measures of puberty.*

**Puberty and cortical thickness correlations with age included**

SEM models that also included chronological age in the model predicting cortical thickness (i.e., thickness ~ puberty + age) are represented visually in Supplemental Figure 4. For comparison, Figure 6 from the main text (correlations without age included) shows that the negative correlations are stronger when age is not included, suggesting that age and cortical thickness share a substantial amount of variance, and hormones especially do not withstand controlling for age when predicting cortical thickness. An additional map of correlations between cortical thickness and chronological age is shown in Supplemental Figure 5.


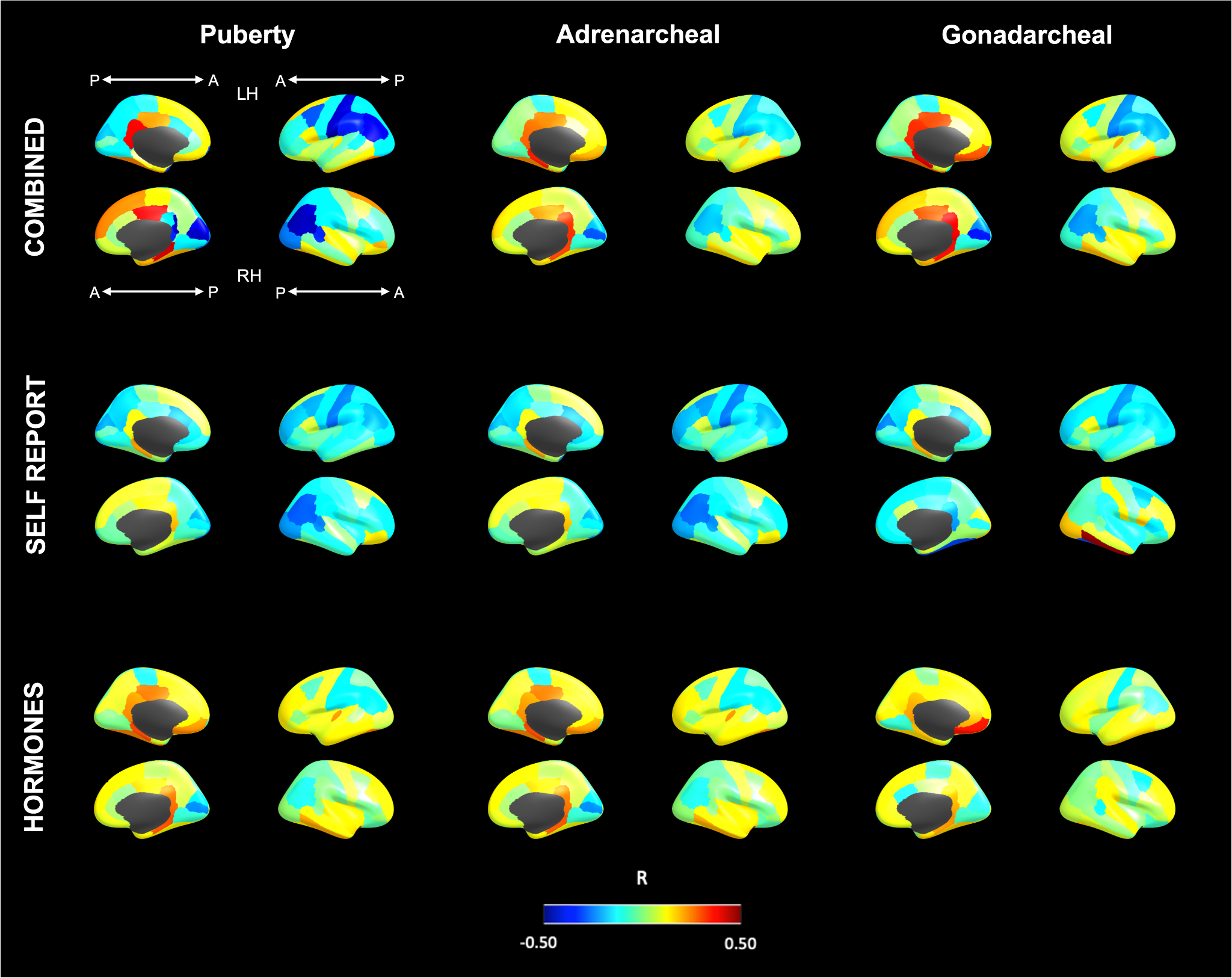


***Supplemental Figure 4****: Estimated correlations from structural equation models between cortical thickness and multi-method (self-reported physical characteristics from the Pubertal Development Scale and Morris & Udry line drawing, and levels of hormone dehydroepiandrosterone, testosterone, and estradiol in saliva) latent factors of overall pubertal, adrenarcheal, and gonadarcheal stage, with chronological age included, in a sample of girls aged 10 - 13. Images were created with freesurfer_statsurf_display ( <https://chrisadamsonmcri.github.io/freesurfer_statsurf_display>). A: anterior; P: posterior; LH: left hemisphere, RH: right hemisphere, R: correlation coefficient estimated in latent models*


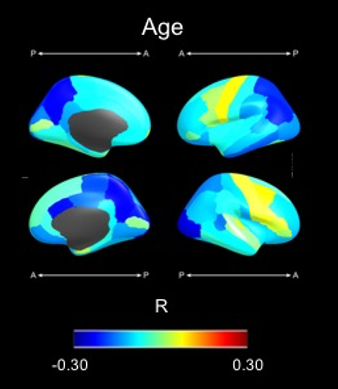


***Supplemental Figure 5****: Pearson correlations between cortical thickness and chronological age, in a sample of girls aged 10 - 13. Images were created with freesurfer_statsurf_display (* [*https://chrisadamsonmcri.github.io/freesurfer_statsurf_display*](https://chrisadamsonmcri.github.io/freesurfer_statsurf_display)*). A: anterior; P: posterior; LH: left hemisphere, RH: right hemisphere, R: correlation coefficient*
